# Potential of transcranial ultrasound- and near-infrared spectroscopy-based acute stroke imaging for decision-making on intravenous thrombolysis treatment

**DOI:** 10.3389/fneur.2025.1499821

**Published:** 2025-02-24

**Authors:** Erik Freitag, Hebun Erdur, Ahmed A. Khalil, Peter Harmel, Maximilian Kaffes, Christoph H. Schmitz, Joachim E. Weber, Heinrich J. Audebert

**Affiliations:** ^1^Department of Neurology, Charité - Universitätsmedizin Berlin, Freie Universität Berlin and Humboldt-Universität zu Berlin, Berlin, Germany; ^2^Center for Stroke Research Berlin, Charité - Universitätsmedizin Berlin, Freie Universität Berlin and Humboldt-Universität zu Berlin, Berlin, Germany; ^3^Hochschule für Technik und Wirtschaft (HTW) Berlin, Berlin, Germany; ^4^DZHK (German Center for Cardiovascular Research), Berlin, Germany; ^5^Berlin Institute of Health at Charité – Universitätsmedizin Berlin, Berlin, Germany

**Keywords:** stroke, intracranial haematoma, TCCS, NIRS (near infrared spectroscopy), MRI, intravenous thrombolysis

## Abstract

**Background:**

Mobile Stroke Units (MSU) shorten time to intravenous thrombolysis (IVT) and improve functional outcome, but they rely on computed tomography (CT) making them highly specialized and costly. Alternative technologies can potentially identify imaging-based IVT contraindications like intracranial hemorrhage (ICH) or malignancies (IM), e.g., by transcranial color-coded sonography (TCCS) and near-infrared spectroscopy (NIRS).

**Methods:**

Using a simulation approach, we analyzed magnetic resonance imaging (MRI) scans of stroke-suspected patients within 4.5 h of symptom onset to assess TCCS and NIRS for identifying imaging-based IVT contraindications. Our study included both primary and sensitivity analyses, each employing conservative and optimistic scenarios. The primary analysis integrated clinical information from the emergency department, while the sensitivity analysis evaluated overall performance across all patients, regardless of clinical information. The conservative scenario defined TCCS detecting acute deep-brain hemorrhages or tumors >20 mm from scalp surface or > 10 mL in volume or causing >4 mm midline-shift, while NIRS was defined detecting them <20 mm from scalp surface with a volume > 3.5 mL. The optimistic scenario defined TCCS detecting intracranial or subarachnoid acute/subacute hematoma or tumors >20 mm from scalp surface or > 5 mL in volume or causing >2 mm midline-shift, while NIRS was defined detecting them <35 mm from the scalp surface with volume > 3.5 mL.

**Results:**

We assessed 1,089 consecutive patients undergoing acute MRI, identifying 69 with imaging-based IVT contraindications, of which 40 had additional non-imaging contraindications. In the primary analysis, among those 29 patients without non-imaging-based contraindications, TCCS/NIRS would have detected 15 of 25 ICH and 3 of 4 malignant tumors in the conservative scenario. In the optimistic scenario, 18 of 25 ICH and all malignant tumors would have been detected. In the sensitivity analyses, the conservative scenario would have detected 30 of 52 ICH and 8 of 17 malignant tumors, while the optimistic scenario would have identified 37 of 52 ICH and 12 of 17 malignant tumors.

**Conclusion:**

While TCCS and NIRS technologies exhibit potential for identifying IVT contraindications in pre-hospital settings, comprehensive evaluation in real-world scenarios is imperative to ascertain their operational constraints.

## Introduction

Before intravenous thrombolysis (IVT) is initiated in acute ischemic stroke (AIS) patients, contraindications for IVT need to be excluded (1). Thus far, only computed tomography (CT) or magnetic resonance imaging (MRI) are used to exclude intracranial hemorrhages (ICH) or malignancies. Until recently, these devices have historically been limited to hospitals, resulting in delayed initiation of specific stroke treatments until hospital arrival. However, in recent years, smaller CT scanners have been installed in specialized stroke ambulances, now referred to as Mobile Stroke Units (MSU) (2). While this approach has demonstrated feasibility, safety, and efficacy in reducing time to treatment (3, 4) and improving functional outcomes in patients with AIS (5–7), it also presents several drawbacks—namely the considerable weight and expense of the scanner as well as extensive additional personnel, procedural and technical infrastructure. The implementation of MSU services is complex, starting with a system for identifying stroke patients at dispatch level (8), as well as maintaining special ambulance vehicles, and establishing comprehensive quality management tailored specifically to the MSU.

The deployment of an alternative CT-independent diagnostic device for stroke on ambulances that is linked via telemedicine could potentially expedite time to stroke treatment on a broad scale. Two promising technologies for this purpose are transcranial color-coded duplex sonography (TCCS) and near-infrared spectroscopy (NIRS). TCCS is well-established for imaging basal brain arteries and can identify ICH in deep brain regions such as the basal ganglia or thalamus (9–12). NIRS, on the other hand, can detect hemorrhages in superficial brain areas, including intracerebral, epi−/subdural and subarachnoid hemorrhage (SAH) (13, 14), and is used to measure changes in oxygenation or perfusion (15–18). However, since neither technology covers the full range of CT imaging in terms of tissue and vessel imaging, only a combined application appears promising for providing sufficient diagnostic accuracy. Considering the limitations in spatial coverage, it remains unclear whether decision-making based on these technologies alone would be safe enough for IVT.

Hence, the aim of this study was to determine the detection rate of imaging-based contraindications to IVT using a combined approach of TCCS- and NIRS in a consecutive cohort of patients undergoing MRI for suspected acute stroke.

## Methods

### Study design and patients

We conducted this simulation study according to a pre-determined study protocol. For the study, we retrospectively analyzed routine data from patients with acute symptoms suspicious for stroke or transient ischemic attack (TIA) who received 3-Tesla-MRI examination as first cranial imaging between 01/2014 and 12/2017. During this period, MRI was the main imaging method for acute stroke assessment at Charité – Campus Benjamin Franklin in Berlin during operation times (Mon-Fri: 7:00 am to 6:00 pm, Sat + Sun: 8:00 am to 12:00 am) in all patients without contraindications to MRI. Each individual patient was recorded in a separate log including information on time from symptom onset to imaging.

The study was approved by the Ethics Committee of the Charité – Universitätsmedizin Berlin (Institutional Review Board number EA1/140/21). Inclusion criteria were MRI imaging undertaken in suspected stroke patients deemed to be potentially eligible for IVT, and age ≥ 18 years. Patients were excluded from the study if fluid-attenuated inversion recovery or T2*-weighted imaging was not completed due to reasons, such as claustrophobia.

### Data collection

Patient enrollment was based on the Neuro-MRI screening log. Additional data were acquired through clinical charts and imaging assessment utilizing REDCap software, a web-based platform developed by Vanderbilt University in Nashville, Tennessee, USA. We extracted data from the hospital information system. The National Institutes of Health Stroke Scale score was used to assess stroke severity. Final hospital diagnosis was based on the International Statistical Classification of Diseases and Related Health Problems, Tenth Revision, [ICD-10]. AIS [ICD-10: I63.x] was coded in patients with acute diffusion weighted imaging (DWI) lesions and with corresponding hypointensity in apparent diffusion coefficient imaging independent of the duration of neurological symptoms. In patients with suspected cerebral ischemia who had negative findings on DWI, the occurrence of AIS was still considered and coded if IVT was administered due to ischemic symptoms. This condition is referred to as neuroimaging-negative cerebral ischemia (NNCI). The diagnosis of stroke mimics (SM) was made based on two important factors: the absence of ischemic lesions on DWI sequences and the presence of an alternate clinical discharge diagnosis (19).

### Imaging acquisition

All patients were examined with a 3 T MR scanner (Magnetom Trio; Siemens AG, Germany, 32-channel head coil) by the MRI unit of the Center for Stroke Research Berlin (CSB) at the Charité Campus Benjamin Franklin. The MRI standard stroke protocol contained DWI including calculated apparent diffusion coefficient maps, T2*-weighted imaging, 3D Time-of-flight-magnetic resonance angiography (MRA) and FLAIR. Detailed information on the MRI sequences can be accessed in the Supplementary material. Patients with suspicion of a brain tumor, either based on clinical assessment or suspicious lesions in other sequences, additionally received a T1-weighted image before and after administration of a gadolinium-based contrast agent (Gadovist, Bayer, Germany). A proportion of these patients also received a contrast-enhanced T1-weighted magnetization-prepared rapid gradient echo sequence (T1-MPRAGE).

Three independent readers evaluated all images: two with over 4 years of neurology training and experience in MRI reading (EF and PH), and one with more than 3 years of experience in MRI neuroradiology (HE). MRI abnormalities were evaluated regarding lesion type (hemorrhagic, ischemic, neoplastic and non-cerebrovascular) and large vessel occlusions. Hemorrhagic and neoplastic lesions were further assessed for location (anatomical structures and shortest distance to scalp), distribution (single/multiple and supra−/infratentorial) and volume. Hemorrhagic lesions were also evaluated for type (intracerebral, subdural, epidural, subarachnoid and intraventricular), and associated midline shift. Age of the ICH (hyperacute, acute, subacute and chronic) was estimated as described by Chalela and Gomes (20).

### Lesion delineation

Lesion volumes of ICH and intracranial malignancies (IM) were delineated manually using MRIcro 1.40 (Chris Rorden, USA). In 44 patients, the lesions were delineated on FLAIR images. In three patients T2* images were employed for measuring. In five patients with SAH (*n* = 4) or intraventricular hemorrhage (*n* = 1), it was not possible to precisely delineate the lesions as they were either too small or otherwise diffusely distributed in the subarachnoid or intraventricular compartments. In two out of seventeen patients with brain tumors already visible in non-contrast FLAIR imaging, no contrast-enhanced T1-weighted image was available during the acute MRI assessment. Tumors were delineated on the B0 image of the DWI or the FLAIR in these cases.

Image processing included brain extraction using the FMRIB Software Library’s Brain Extraction Tool [FSL BET (21)]. The images and delineated ROIs were then nonlinearly registered to the Montreal Neurological Institute T1-weighted template (MNI152 2 mm) using Advanced Normalization Tools [ANTs (22)]. The registered ROIs were then binarized, concatenated, and summed-up to create a heat map of ROI location.

### Assessment of diagnostic detectability

A model for lesion detectability by TCCS and NIRS was developed based on the limitations of each technique, as reported in the literature. The model considered two scenarios, conservative and optimistic, each using different criteria for lesion type, location, sonographically accessible areas, volume, age (as defined by MRI criteria), midline shift and detectability of brain tumors. The conservative scenario employed only criteria that have been consistently reported in the literature, while the optimistic scenario relied on inconsistently reported criteria. The derivation of assumptions for each scenario are detailed in the Supplementary material, and Table 1 summarizes the relevant criteria.

**Table 1:** Assumptions for the scenarios.

Criteria for detection	NIRS	TCCS
Conservative scenario		
Distance to scalp surface	<20 mm	>20 mm
Region	Supratentorial	Supratentorial except fronto-, occiptopolar, parasagittal and infratentorial regions
ICH volume	>3.5 mL	>10 mL
Lesion age according to MRI criteria	Acute	Acute
Midline shift	n.a.	>4 mm
Detection of brain tumors	no	yes
Detection of SAH	no	no
Optimistic scenario		
Distance to scalp surface	<35 mm	>20 mm
Region	Supratentorial	Supra- and infratentorial (to the lower pontine level) except fronto-, occipitopolar, parasagittal regions
ICH volume	>3.5 mL	>5 mL
Lesion age according to MRI criteria	Acute and subacute	Acute and subacute
Midline shift	n.a.	>2 mm
Detection of brain tumors	no	yes
Detection of SAH	yes	no

### Statistical analysis

For descriptive analyses, we utilized IBM SPSS Statistics 27, and reported the results as means with standard deviations (SD) and medians with interquartile ranges (IQR). Furthermore, we utilized an Excel worksheet to assess the detectability of ICH or brain tumor in patients and to calculate sensitivity values for each scenario.

We conducted a primary and a sensitivity analysis. In the primary analysis, we incorporated clinical information from the emergency department to reflect typical clinical decision-making. This included factors such as time from symptom onset or last-seen-well to imaging, presence of neurological symptoms with or without detectable abnormalities, epileptic seizure at the time of symptom manifestation, known malignancy with possible cerebral metastasis, thunderclap headache suggesting SAH, use of direct oral anticoagulants or low-molecular-weight heparin, and history of recent head trauma. After excluding these IVT contraindications, the remaining patients formed the primary population, in which we performed the primary analysis.

In the sensitivity analysis, we evaluated the overall performance in identifying imaging contraindications for IVT across all patients in the study, without restricting the total patient population based on clinical information.

## Results

### Baseline characteristics

**Figure 1:**
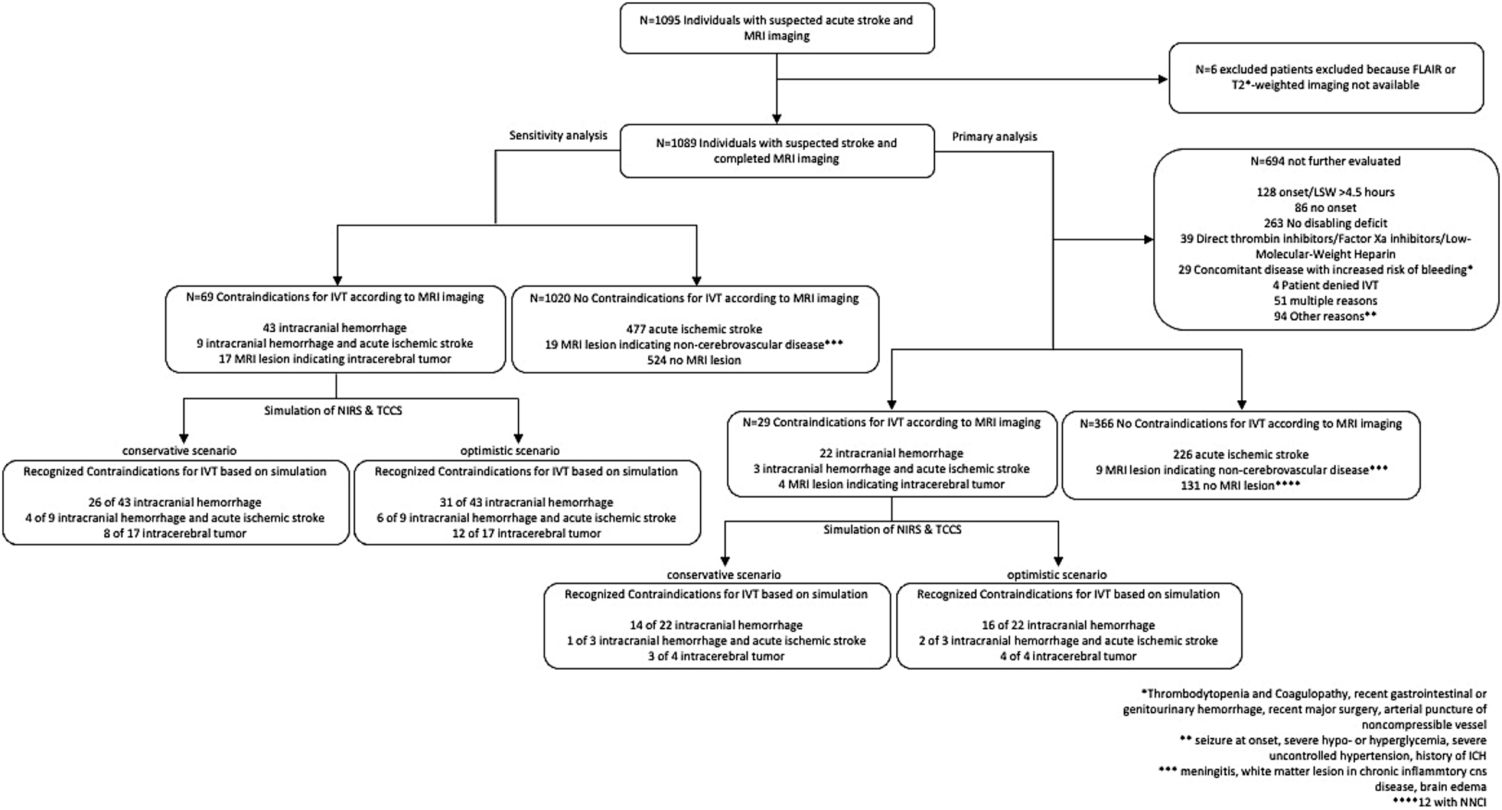
Participant enrollment.

We enrolled 1,089 consecutive patients with clinical symptoms suggestive of acute stroke, who underwent MRI imaging within 4.5 h of symptom onset or last-seen-well and were admitted to the stroke unit or intensive care unit (see Figure 1). Out of these patients, 214 were initially suspected to have a time window within 4.5 h, which had to be corrected to a greater than 4.5-h time window or an unknown onset during further examination. This reflects the difficulty in accurately determining the exact onset of symptoms, particularly in patients with aphasia. Furthermore, 480 patients had non-imaging-based contraindications against IVT. After excluding patients with these contraindications, the primary population consisted of 395 patients who were potentially eligible for IVT. The mean age of this group was 70.6 years [standard deviation (SD): 15.9 years], with 54.2% of them being female. TCCS was performed in 224 of the 395 patients (see Table 2). Sufficient temporal bone windows were identified in 75.4% of these cases. TCCS was not performed in all patients based on clinical decision-making, e.g., in case of SM or ICH, with no need for additional vessel imaging.

**Table 2:** Baseline characteristics of the primary population (primary analysis).

	All patients	Ischemic stroke	Intracranial hemorrhage	Ischemic stroke and intracranial hemorrhage	MRI lesions indicating non-cerebrovascular disease	No MRI lesion
	*N* = 395	*N* = 226	*N* = 22	*N* = 3	*N* = 13	*N* = 131
*Age, mean (SD), y*	70.6 (15.9)	74.4 (12.7)	77.4 (11.7)	77.0 (11.4)	68.6 (14.2)	62.9 (18.7)
*Sex*						
Female, N (%)	214 (54.2)	123 (54.4)	17 (77.3)	0 (0)	7 (53.8)	67 (51.1)
Initial NIHSS, median (IQR)	7 (3–13)	7 (3–14)	11 (5.75–14)	8 (3–8)	5 (0–5)	3 (1–6)
Missings, N (%)	131 (33.2)	20 (8.8)	0 (0.0)	0 (0.0)	10 (76.9)	101 (77.1)
Time from onset or last-seen well ≤4.5 h to imaging, median (IQR), min	107 (74–162)	96 (70–142)	91 (61–147)	148 (148–162)	168 (97–205)	131 (83–175)
Sufficient temporal bone window for TCCS	169/224 (75.4)	145/195 (74.4)	2/2 (100)	3/3 (100)	2/3 (66.7)	17/21 (81)
Denied TCCS examination, N (%)	4 (1)	4 (1.8)				
TCCS not performed, N (%)	167 (42.3)	27 (12)	20 (91)		10 (77)	110 (84)
Treatment with intravenous thrombolysis, N (%)	243 (61.5)	226 (100)	0 (0.0)	0 (0.0)	1 (7.7)	16 (12.2)

Tables 2, 3 provide baseline characteristics of the primary population and the total population, as well as subgroups of (a) patients with AIS, (b) ICH, (c) combined ischemic and hemorrhagic MRI lesions, (d) non-cerebrovascular disease, (e) and patients without MRI lesions.

**Table 3:** Baseline characteristics of the total population (sensitivity analysis).

	All patients	Ischemic stroke	Intracranial hemorrhage	Ischemic stroke and intracranial hemorrhage	MRI lesions indicating non-cerebrovascular disease	No MRI lesion
	*N* = 1,089	*N* = 477	*N* = 43	*N* = 9	*N* = 36	*N* = 524
*Age, mean (SD), y*	70.7 (15.3)	74.3 (12.8)	76.4 (10.0)	77.9 (10.9)	64.7 (14.7)	67.3 (16.8)
*Sex*						
Female, N (%)	595 (54.6)	247 (51.8)	30 (69.8)	4 (44.4)	23 (63.9)	291 (55.5)
Initial NIHSS, median (IQR)	4 (1–9)	5 (2–11)	10 (5–14)	6.5 (3.25–13)	1 (1–5)	1 (0–3)
Missings, N (%)	478 (43.9)	85 (17.8)	0 (0.0)	1 (11.1)	29 (80.5)	363 (69.3)
Time from onset or last-seen well ≤4.5 h to imaging, median (IQR), min	132.5 (83–216)	122 (77–226)	96.5 (69.75–154.259)	155 (118.5–516.75)	163 (91.75–235)	140 (93–211)
Last seen well ≥270 min, N (%)	150 (13.8)	91 (19.1)	4 (9.3)	2 (22.2)	4 (11.1)	49 (9.3)
Missings (N)	102	25	3	1	4	69
Sufficient temporal bone window for TCCS, N / number of examined patients (%)	468/624 (75)	293/404 (73)	5/5 (100)	6/6 (100)	5/6 (83.3)	159/203 (78.3)
Denied TCCS examination, N (%)	10 (1)	9 (1.9)				1 (0.2)
TCCS not performed, N (%)	455 (41.8)	64 (13.4)	38 (88.4)	3 (33.3)	30 (83.3)	320 (61.1)
Clinical IVT contraindication, N (%)	694 (63.7)	251 (52.6)	21 (55.8)	6 (66.7)	23 (64)	393 (75)
Treatment with intravenous thrombolysis, N (%)	276 (25.3)	259 (54.3)	0 (0.0)	0 (0.0)	1 (2.8)	16 (3.1)

A subgroup of 524 patients of the total population did not exhibit MRI lesions. Of these patients, 33 were diagnosed with AIS, 121 with TIA, and 370 with other conditions, such as epileptic seizures (47 patients), vertigo (87 patients, of whom 45 had peripheral vertigo), migraine (35 patients), and transient global amnesia (15 patients). Among the primary population, 131 had no MRI lesion of whom 119 had a SM. Seizures were the most common SM (24%), followed by migraine (21%).

Among the total population, 276 patients received IVT, of whom 259 were diagnosed with AIS, 12 with NNCI, and 5 with non-cerebrovascular diagnoses. Thirty-three of these patients had an unknown symptom onset and were deemed eligible for IVT based on MRI criteria for wake-up stroke (DWI-FLAIR mismatch). In the primary population of 243 patients who received IVT, 226 were diagnosed with AIS, 12 with NNCI, and 5 with non-cerebrovascular diagnoses.

ICH were identified in 52 patients, with 3 of them being located in the posterior fossa, specifically 1 pontine and 2 cerebellar hematomas. Comprehensive details regarding the characteristics of all ICH and IM can be found in the supplement. Additionally, 36 patients were found to have intracranial non-cerebrovascular lesions, with 17 of them being brain tumors (3 of these tumors being benign (meningioma), not considered as contraindications against thrombolysis). However, these tumors may have led to a decision against thrombolysis in non-CT/MRI-based brain imaging. Out of the 69 patients who were deemed ineligible for IVT based on imaging findings, 40 patients also had non-imaging contraindications. These included severe head trauma prior to hospital admission (*N* = 6), a recent diagnosis of IM or recent brain surgery (*N* = 1), use of oral anticoagulation (*N* = 5), unknown time of onset (*N* = 5), status epilepticus (*N* = 2), known malignancy (*N* = 9) or multiple reasons (including one SAH patient who presented with sudden onset headache) (*N* = 12).

**Figure 2:**
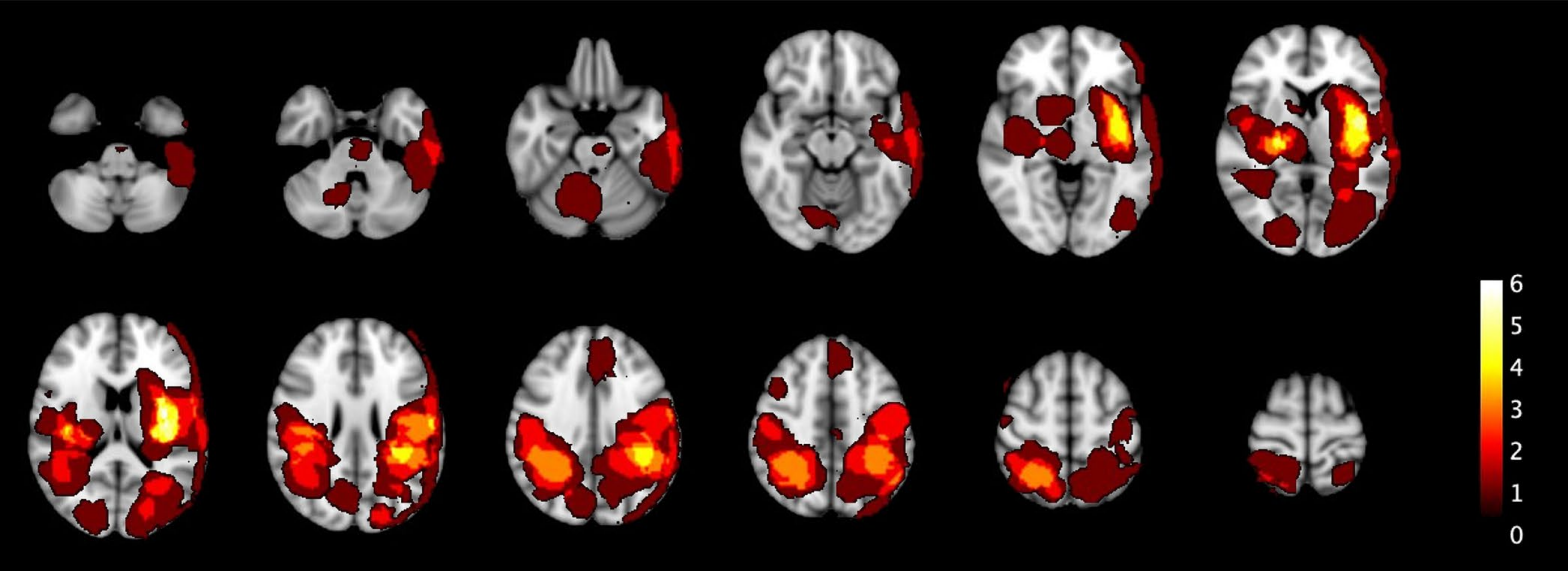
The heatmaps visualize the spatial distribution of all 25 ICH and 4 IM across different sections of the brain in the primary population. The color scale represents lesion frequency, with brighter colors indicating higher densities. The highest concentrations are observed in the deep basal ganglia, parietal lobes, and parieto-occipital regions. These heatmaps provide insights into the lesion locations, helping to understand their spatial distribution within the studied cohort.

**Figure 3:**
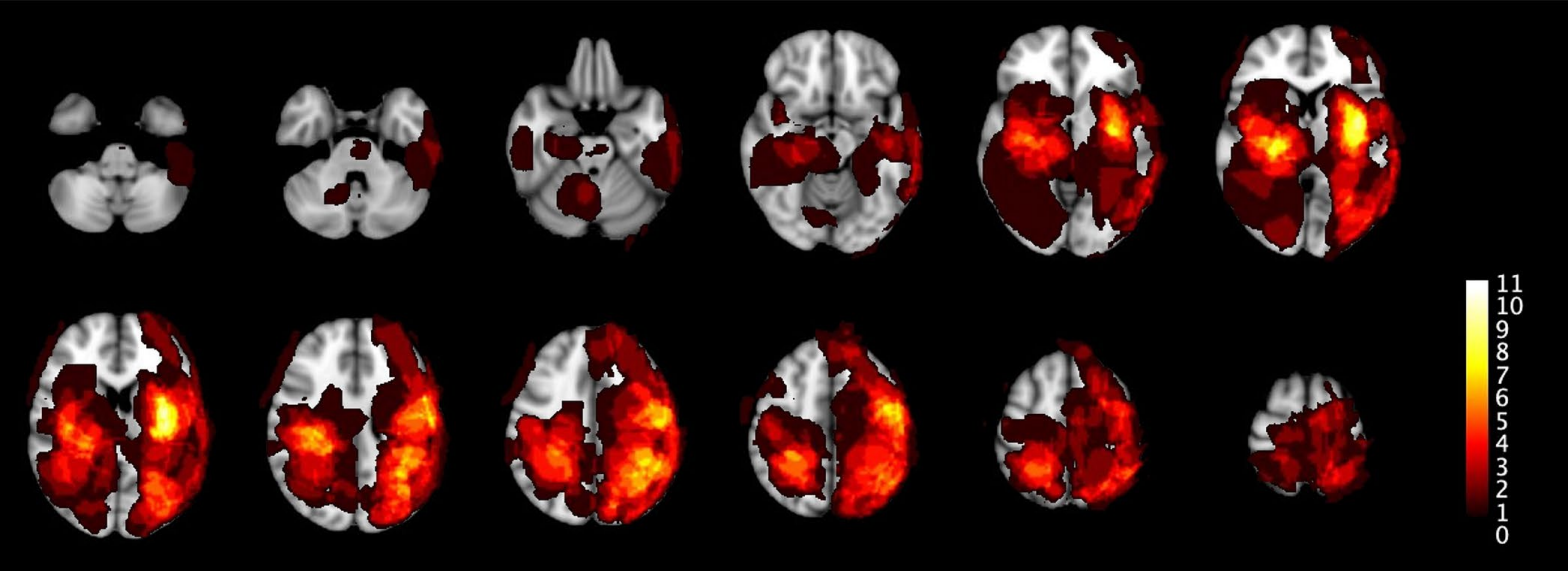
This figure extends the analysis from Figure 2 to the total population. The distribution patterns remain similar but reflects the larger dataset including all 52 ICH and 17 IM.

Figure 2 shows the distribution of all the ICH and IM in the primary population and Figure 3 of the total population.

### Primary analysis

**Figure 4:**
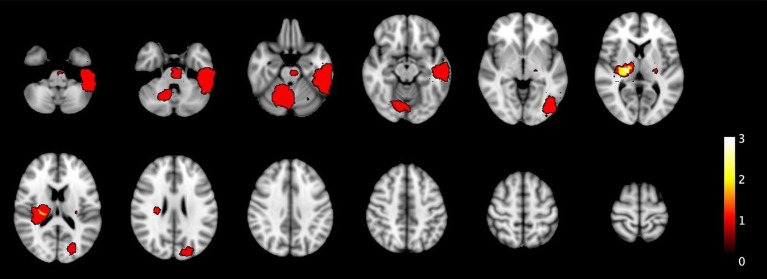
The heatmaps displays the spatial distribution of undetected ICH and IM in the primary population that were not detected by TCCS and NIRS in the conservative scenario. One brain tumor was undetected, located in the left temporal lobe, while all other lesions are ICH smaller than 10 mL. Additionally, two ICH are located in the pons and right cerebellar region.

**Figure 5:**
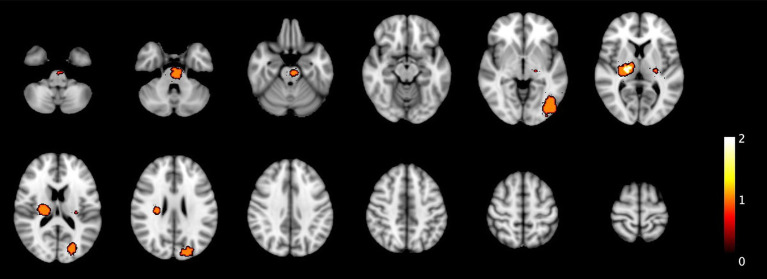
Similar to Figure 4, this figure presents the undetected ICH under the primary population for the optimistic scenario.

In the conservative scenario, 15 out of 25 ICH and 3 out of 4 malignancies would have been detectable by at least one of the diagnostic modalities, with 6 being detected by both TCCS and NIRS, 9 being detected by TCCS B-mode and 3 being detected by midline shift only. However, 10 cases of ICH and one case of IM would have gone undetected. Notably, 4 of these cases presented with very mild symptoms, which is a relative contraindication to IVT. In the optimistic scenario, all 4 cases of IM and 18 cases of ICH would have been detected, with 13 cases being detected by both TCCS and NIRS, 7 by TCCS B-mode and 1 by midline shift only, and 1 by NIRS only (Table 4). All undetectable 7 cases of ICH had volume sizes of less than 3 milliliters. Figure 4 illustrates the location of the ICH and one IM which in the conservative scenario would not have been detected, while Figure 5 displays all ICH that would have been missed in the optimistic scenario.

**Table 4:** Results of primary and sensitivity analysis for ICH and malignant tumors with TCCS/NIRS.

	Conservative scenario	Optimistic scenario
Primary analysis		
ICH	15/25 (60%)	18/25 (72%)
IM	3/4 (75%)	4/4 (100%)
ICH and IM	18/29 (62%)	22/29 (76%)
Sensitivity analysis		
ICH	30/52 (58%)	37/52 (71%)
IM	8/17 (47%)	12/17 (71%)
ICH and IM	38/69 (55%)	49/69 (71%)

### Sensitivity analysis

In the conservative scenario, 30 out of 52 ICH would have been detected, along with 8 out of 17 IM. Of the 30 ICH that were detected, 13 would have been identified by both TCCS and NIRS, 14 by TCCS alone, and 3 by midline shift.

In the optimistic scenario, 37 out of 52 ICH would have been detected, as well as 12 out of 17 IM (Table 4). Of the 37 ICH that were detected, 22 would have been identified by both TCCS and NIRS, 8 were detected by TCCS B-mode and 4 were detected by midline shift, and 3 were detected by NIRS (2 subarachnoid bleeds and 1 subdural bleeding).

## Discussion

In our sample of 1,089 consecutive suspected acute stroke patients undergoing MRI imaging, 5% had ICH and 2% had IM. In primary analysis that incorporated clinical information, conservative and optimistic scenarios detected 62 and 76% of ICH and IM. For timely imaging, sensitivity analysis would have found 55 and 71% sensitivity, highlighting greater uncertainty in detecting ICH and IM in patients seen beyond 4.5 h or with unknown time of onset.

Assuming no technical feasibility limitations and with IVT administration to all eligible patients, we extrapolate that in the conservative scenario, a total of 254 patients with AIS would have received prehospital IVT, including 11 patients with missed imaging-based contraindications identified by TCCS and NIRS. In the optimistic scenario, 250 patients would have received IVT. Furthermore, utilizing TCCS and NIRS would have led to providing treatment to a larger number of patients, including those 131 patients with disabling deficits who were deemed ineligible for MRI-based thrombolytic procedures due to the absence of DWI lesions. The majority of them was ultimately diagnosed as SM. Several studies have shown that patients with SM do not exhibit a critical risk of adverse events from thrombolytic therapy (23, 24). Although there is a paucity of literature on outcomes of patients who receive IVT despite having hemorrhagic lesions on baseline imaging, it is highly likely that ICH would result in worse outcomes after IVT. While stroke imaging based on TCCS and NIRS may not detect all ICH, it offers a valuable tool for identifying eligible candidates for IVT. However, the outcome of patients with very small baseline ICH who receive IVT requires further investigation. If the rate of undetected ICH or IM remains low, the benefits of administering IVT more quickly (i.e., in prehospital settings) may outweigh the increased risk of hemorrhagic complications.

Recently published large prehospital studies in Berlin and the United States indicate that out of 100 stroke patients without contraindications for IVT, prehospital treatment resulted in 9 or 10 additional patients surviving without disability (measured by the modified Rankin Scale 0 or 1) and 2 or 3 fewer patients dying. In our study, with 243 patients who received IVT in the primary population, using the same proportion of tPA treatments as in the Berlin B_PROUD study (5) suggests that prehospital treatment could have resulted in approximately 24 fewer patients with disability and 5 fewer deaths. Under the assumption that all undetected ICH or IM in the conservative scenario would have been fatal, 6 more patients would have died, while in the optimistic scenario mortality would have remained more or less unchanged. These projections do not account for additional benefits of accurate routing to thrombectomy-capable centers based on TCCS vessel imaging.

A potential enhancement of the safety profile of the aforementioned diagnostic approach could be achieved by leveraging the supplementary functionalities of TCCS-based vessel and perfusion imaging in conjunction with NIRS-based perfusion measurement. When the observed clinical symptoms align with the region affected by an occluded artery or a perfusion deficit evident in a specific perfusion territory, the probability of a concurrent ICH becomes notably low. In our sample, such coincidence only occurred twice. In one instance, a patient being on oral anticoagulation experienced left-sided hemianopia, had a right posterior cerebral arteria occlusion, and a large subacute subdural hematoma with a midline shift of 0.3 mm. In the second instance, a patient experienced sudden left-sided hemiparesis and was found to have a chronic occlusion of the right internal carotid artery, as well as an intraventricular hemorrhage and midline shift of 0.6 mm. In both cases, TCCS would have yielded a contraindication by detecting the midline shift. Additionally, the use of perfusion imaging with both ultrasound and NIRS may increase the sensitivity of identifying densely vascularized brain tumors.

Like all simulation studies, there are numerous uncertainties that require a step-by-step process beginning with the creation of a technically stable diagnostic tool that can quickly assess patients and produce the intended outcomes. Subsequently, the diagnostic accuracy of the tool must be verified against the gold standard, which is CT imaging, ideally in MSUs. Compared to CT scanners, even a combination of both bilateral TCCS devices and a NIRS device is far more compact and thus can fit into every standard ambulance. A workflow with a similar duration as a CT scan appears feasible if robotic-controlled and AI-assisted. If clinical decision-making based on the results of combined alternative diagnostic technologies proves to be as reliable as CT-based brain imaging, then a controlled and preferably randomized trial should be conducted to investigate the impact on outcomes in standard (telemedicine enhanced) ambulances.

A strength of our study is that the simulation was conducted on a large sample of consecutive patients with suspected acute stroke, including those with final non-cerebrovascular diagnoses. However, there are several limitations. First, selection bias is introduced as only suspected stroke patients who underwent MRI imaging were included. At our institution, patients with unstable vital parameters or acute loss of consciousness typically receive CT imaging as their first imaging modality. This could result in an underrepresentation of large hematomas that are in reverse more likely to be detected by TCCS or NIRS. The percentage of ICH in our study (5%) was lower than the rates observed in our hospital during the study period ranging from 8.2 to 12.3%. Patients with motor restlessness are not suitable for MRI, and therefore, they may have undergone CT imaging instead. However, motor restlessness is also equally likely to be a contraindication for the alternative technologies, particularly TCCS, and as a result, such patients may not be eligible for prehospital assessment by such technologies. Automated or robotic use becomes more crucial to enable examination in the absence of skilled personnel or under difficult conditions. Second, the technologies utilized in this simulation study have not yet been evaluated for the detection of ICH or IM in the prehospital setting. Due to the variability of diagnostic accuracy reported in the literature, we were cautious in making assumptions for both scenarios, particularly in the conservative scenario. However, future advancements in these technologies may lead to improved diagnostic capabilities, particularly when combined with artificial intelligence-assisted algorithms for analyzing multisource data. Third, a considerable proportion of patients do not exhibit an adequate temporal bone window for TCCS, reducing the feasibility of prehospital stroke differentiation. Finally, our analysis, which emphasizes the identification of ICH or tumor detection, was based on findings reported in the current literature. Since these assessments were frequently carried out in restricted patient populations, we cannot provide reliable estimates of the specificity and positive predictive rates of TCCS and NIRS in unselected populations. Therefore, these measures must be evaluated in standard prehospital patient cohorts with suspected stroke. For instance, TCCS may misdiagnose intracranial calcifications and the choroid plexus hemorrhages.

## Conclusion

The combination of TCCS and NIRS holds promise for identifying most imaging-based contraindications to IVT. Utilizing these technologies in prehospital stroke workup could result in significant improvements in acute stroke outcomes, given the substantial impact of IVT in the hyperacute phase within 60 min of symptom onset (25), but will need widely automated utilization of such devices. Further investigation is necessary through real-world studies that compare the diagnostic accuracy of the combined approach with CT-based imaging in MSUs.

## Data Availability

The original contributions presented in the study are included in the article/Supplementary material, further inquiries can be directed to the corresponding author.
