# Supplementary material for: Potential of transcranial ultrasound- and near-infrared spectroscopy-based acute stroke imaging for decision-making on intravenous thrombolysis treatment
Source: Front Neurol. 2025 Feb 24;16:1499821. doi: 10.3389/fneur.2025.1499821 (PMC11891060; doi:10.3389/fneur.2025.1499821)
Supplement: Supplementary file 1 [file Supplementary_file_1.docx]

**Supplemental Material – Frontiers in Neurology**

**Potential of transcranial ultrasound- and near-infrared spectroscopy-based acute stroke imaging for decision-making on intravenous thrombolysis treatment**

Erik Freitag (MD), Hebun Erdur (MD), Ahmed A Khalil (MD PhD), Peter Harmel (MD), Maximilian Kaffes (MD), Christoph H. Schmitz, Joachim E. Weber (MD), Heinrich J. Audebert (MD)

**Corresponding Author:**

Erik Freitag

Department of Neurology, Charité – Universitätsmedizin Berlin, Campus Benjamin Franklin

Hindenburgdamm 30, 12203 Berlin, Germany

Phone +49-30-8445 2276, FAX +49-30-84454264, E-Mail: erik.freitag@charite.de

**Table of Content:**

Derivation of a TCCS-NIRS model based on published data

Detailed information on the MRI sequences

eTable 1: Characteristics of all intracranial hemorrhages and malignancies

eTable 2: Missed ICH and IM in the primary population for both the conservative and optimistic scenarios

Supplemental References

**Derivation of a TCCS-NIRS model based on published data**

Lesion volume

The ability to visualize ICH using TCCS and NIRS depends on the size of the hemorrhage volume. Seidel and Kukulska et al reported detection of hemorrhages as small as 0.5ml using TCCS in their studies^1,2^. However, achieving such high-resolution ultrasound imaging does not appear realistic in a prehospital setting with motion artefacts, noise, and suboptimal lighting conditions. To address this issue, we established specific volume thresholds for hemorrhage detection in each scenario. As a simplified assumption, we used thresholds for the reported of 0,5 ml lesion volumes multiplied by a factor 20 for the conservative scenario and factor of 10 for the optimistic scenario, resulting in a minimum detection volume of 10ml and 5ml, respectively.

Robertson et al. conducted a double blind study for FDA clearance across five different clinical sites, demonstrating with NIRS detection of bleeding was associated with hematoma volume within the brain.^3^ They reported high sensitivity (88%) for detecting hematomas greater than 3.5ml and 100% sensitivity for lesions larger than 70ml. Therefore, we used 3.5ml as our threshold for detecting cerebral hemorrhages in both scenarios for NIRS. In the same study, they detected SAH in 9 out of 13 patients using NIRS. As a result, we defined SAH as detectable only in the optimistic scenario.

Lesion location

Due to the limited ability to image certain areas with transtemporal TCCS, we identified certain regions, such as fronto- and occipitopolar and parasagittal areas, as non-insonable areas for TCCS in both scenarios^1^. Kukulska et al reported 100% sensitivity in detecting infratentorial lesions using TCCS^2^. Due to limited data in visualizing these areas, we only considered infratentorial hemorrhages to the lower pontine level as detectable in the optimistic scenario.

The depth sensitivity profile of NIRS to brain tissue is related to the distance between the source and detector due to the absorption and scattering of photons. Using a phantom model and Monte Carlo simulation, Strangman et al showed that NIRS can penetrate up to 15mm into the intracranial space.^4^ In the aforementioned double-blinded clinical study, Robertson et al. reported high sensitivity (88%) in detecting brain hematomas of up to 25mm from brain surface using a portable NIRS device called Infrascanner.^3^ Based on these findings, and assuming a mean thickness of the scalp, skull and cerebrospinal fluid (CSF) layers of at least 10mm^4–6^ we defined detectability of intracranial hemorrhages (including SAH) within a distance of less than 20mm below the scalp surface for the conservative NIRS scenario and less than 35mm below the scalp surface for the optimistic scenario.

Another limitation of NIRS is its inability to detect lesions located within the infratentorial region. This limitation is primarily due to the increased thickness of the layer above the skull, which impedes the near-infrared light from reaching the intracranial compartment, with the neck muscles being the main cause. We have assumed in both scenarios that NIRS is unable to detect infratentorial lesions.

The fundamental principle underlying hemorrhage detection with NIRS is the measurement of an increase in optical density and hence the reduction of transmitted light. Compared to normal brain tissue where blood is only contained within the blood vessels, the volume concentration of hemoglobin is significantly higher in brain hemorrhages, leading to increased light absorption. To account for inter-subject variability and the method’s inability to accurately measure absolute hemoglobin concentration, lesions are revealed by relative differences when comparing measurements between the hemispheres, which yield difference in optical density (ΔOD)^7^. Because the method relies on hemispheric asymmetries in the measurements, bilateral hemorrhages in the same location cannot be reliably detected by NIRS, and this limitation was included in our scenarios.^8^

Lesion age

As a result of the breakdown of red blood cells following a cerebral hemorrhage, the sonographic appearance undergoes changes over time with decreasing contrast over time.^1^

For NIRS, hemoglobin is the primary light absorber in biological tissue.^9^ After cerebral hemorrhage, the initial hemoglobin concentration decreases over the course of hours to days due to scavenging mechanisms, thereby decreasing light absorption, leading to a decrease in the detectability of subacute and chronic hemorrhage. Thus, in our conservative scenario, we have limited the detection of acute bleedings for both techniques, TCCS and NIRS. In the optimistic scenario, we assumed that both techniques can detect subacute hemorrhages, while chronic hemorrhage were deemed undetectable.

Midline shift (MLS)

Within a 4.5 hour time frame, acute cerebral infarction does not produce space-occupying effects.^10^ Consequently, MLS of the brain is suggestive of intracranial hemorrhage or tumor, which are contraindications for IVT. To address the measuring inaccuracies of TCCS, we established cutoff values. In a recent systematic review, Hakim et al demonstrated that TCCS is a reliable diagnostic tool for evaluating MLS in adults.^11^ They observed a high level of agreement between TCCS and CT, with MLS measurements raging from -1.22 to 0.16mm compared to corresponding CT values. Seidel et al. studied 10 healthy volunteers and identified a mean midline dislocation of 0.2 +/-0.3 mm.^12^ Kukulska-Pawluczuk et al. compared the findings of TCCS and CT in 34 patients with spontaneous ICH. The MLS measured by TCCS ranged from 0.9-20.8 mm.^2^ To account for measuring inaccuracies in the prehospital setting, we used a factor of 20 for the smallest detectable reported MLS reported for the conservative scenario and a factor of 10 for the smallest MLS for the optimistic scenario, resulting in detection thresholds greater than 4 and 2mm, respectively.

Detection of brain tumors

Becker et al. conducted a series of studies in the early 1990s to assess the efficacy of TCCS in detecting intracranial neoplasms.^13,14^ Their findings indicated that the sensitivity for detecting tumors using B-mode imaging was between 80-96%, compared to primary imaging with CT. However, a review by van Leyen et al. in 2011 identified several reasons for non-detection, including insufficient bone window, tumor location outside the possible insonation regions of TCCS, and isoechogenicity in comparison to adjacent tissue in low-grade gliomas.^15^ Despite our efforts, we were unable to find valid data for tumor detection using NIRS. Therefore, we decided to use the same criteria for tumor detection as for brain hemorrhage detection with TCCS in both scenarios. Given the lack of data, we concluded that tumors are not detectable by NIRS in either scenario in our model.

**Detailed information on the MRI sequences**

- Diffusion weighted imaging (DWI): slice thickness 2.5 mm, repetition time TR 8900 ms, echo time TE 93 ms, slice gap 0%, b values were 0 and 1000mm*2/s, 6 directions
- T2*-weighted imaging: slice thickness 5 mm, TR 620 ms, TE 20 ms, slice gap 10%
- 3D Time-of-flight (TOF)-magnetic resonance angiography (MRA): slice thickness 0.7 mm, TR 22 ms, TE 3.86 ms, 27.5% overlapping slices gap 27.5%, flip angle 18°
- Fluid-attenuated inversion recovery (FLAIR): slice thickness 5 mm, TR 8000 ms, TE 100 ms; slice gap 0%
- T1-weighted image: TR = 200 ms, TE = 2.46 ms, flip angle = 70, voxel size = 0.7 x 0.7 x 5 mm
- T1-weighted magnetization-prepared rapid gradient echo sequence (T1-MPRAGE): TR = 1900 ms, TE = 2.52 ms, inversion time = 900 ms, flip angle = 9, voxel size = 1 x 1 x 1 mm)

**eTable 1: Characteristics of all intracranial hemorrhages and malignancies**

|  | Intracranial hemorrhages | intracranial malignancies |
| --- | --- | --- |
|  | N=52 | N=17 |
| *Age, mean (SD), y* | 76.7 (10.0) | 65.2 (13.4) |
| *Sex* |  |  |
| Female, N (%) | 34 (65.4) | 10 (58.8) |
| Initial NIHSS, median (IQR) | 9 (4-14) | 1 |
| Missings, N | 1 | 16 |
| Time from onset or last seen well to imaging, median (IQR), min | 103 (74-162) | 147 (74-212) |
| Missings (N) | 4 | 2 |
| Radiological findings, N (%) |  |  |
| Distribution |  |  |
| single | 42 (80.8) | 11 (64.7) |
| multilocular | 10 (19.2) | 6 (35.3) |
| supratentorial | 47 (90.4) | 14 (82.4) |
| infratentorial | 3 (5.8) | 0 |
| supra- and infratentorial | 2 (3.8) | 3 (17.6) |
| right | 23 (44.2) | 0 |
| left | 26 (50) | 10 (58.8) |
| both sides | 3 (5.8) | 7 (41.2) |
| Type of ICH, N (%) |  |  |
| intracerebral | 42 (80.8) | n.a. |
| subdural | 5 (9.6) | n.a. |
| subarachnoid | 4 (7.7) | n.a. |
| epidural | 0 (0) | n.a. |
| intraventricular | 1 (1.9) | n.a. |
| Age of ICH, N (%) |  |  |
| acute | 44 (84.6) | n.a. |
| subacute | 6 (11.5) | n.a. |
| chronic | 2 (3.8) | n.a. |
| Volume, mean (SD), ml | 28.4 (33.8)* | 24.2 (21.5)** |
| Distance to brain surface, mean (SD), mm | 2.7 (1.5) | 2.11 (1.0) |
| Midlineshift, N (%) | 16 (30.8) | 5 (29.4) |
| >4 mm | 11 (21.2) | 2 (11.8) |
| >2 mm | 16 (30.8) | 5 (29.4) |
| Intraventricular extension^#^, N (%) | 11 (21.2) | 0 |
| Presumed ICH etiology |  |  |
| hypertensive | 15 (28.8) | n.a. |
| traumatic | 4 (7.7) | n.a. |
| CAA assoiciated | 10 (19.2) | n.a. |
| aneurysm | 1 (1.9) | n.a. |
| congestive bleeding | 2 (3.8) | n.a. |
| cavernoma associated | 1 (1.9) | n.a. |
| arterio-venous malformation | 2 (3.8) | n.a. |
| tumor associated | 0 (0) | n.a. |
| pharmatoxocological | 0(0) | n.a. |
| others | 8 (15.4) | n.a. |
| unknown | 9 (173) | n.a. |

*volumetrics based on FLAIR-images, **volumetrics based on T1-post KM-images, ^#^ filling of >50% of one ventricle

**eTable 2: Missed ICH and IM in the primary population for both the conservative and optimistic scenarios**

| **primary population conservative scenario** | | | | | | |
| --- | --- | --- | --- | --- | --- | --- |
| Case No. | Pathology | Distance to scalp surface (mm) | Volume (ml) | MLS > 4mm | Insonable area | Age of lesion |
| 4 | ICH | 57,20 | 3,05 | no | no | acute |
| 9 | ICH | 52,00 | 2,58 | no | yes | acute |
| 13 | ICH | 22,10 | 2,25 | no | no | acute |
| 17 | ICH | 65,00 | 0,55 | no | yes | acute |
| 26 | ICH | 54,10 | 0,47 | no | yes | acute |
| 29 | ICH | 28,30 | 12,38 | no | no | acute |
| 30 | ICH | 51,00 | 2,09 | no | yes | acute |
| 31 | ICH | 11,40 | 2,60 | no | yes | acute |
| 38 | ICH | 44,10 | 7,26 | no | yes | acute |
| 43 | SAH | 14,10 | n.a. | no | no | acute |
| 56 | Astroglioma | 14,50 | 8,29 | no | yes | n.a. |
|  |  |  |  |  |  |  |
| **primary population optimistic scenario** | | | | | | |
| Case No. | Pathology | Distance to scalp surface (mm) | Volume (ml) | MLS > 2mm | Insonable area | Age of lesion |
| 4 | ICH | 57,20 | 3,05 | no | yes | acute |
| 9 | ICH | 52,00 | 2,58 | no | yes | acute |
| 13 | ICH | 22,10 | 2,25 | no | no | acute |
| 17 | ICH | 65,00 | 0,55 | no | yes | acute |
| 26 | ICH | 54,10 | 0,47 | no | yes | acute |
| 30 | ICH | 51,00 | 2,09 | no | yes | acute |
| 31 | ICH | 11,40 | 2,60 | no | yes | acute |

Supplemental References

1. Seidel G, Kaps M, Dorndorf W. Transcranial color-coded duplex sonography of intracerebral hematomas in adults. *Stroke*. 1993;24(10):1519-1527.

2. Kukulska-Pawluczuk B, Ksiazkiewicz B, Nowaczewska M. Imaging of spontaneous intracerebral hemorrhages by means of transcranial color-coded sonography. *Eur J Radiol*. 2012;81(6):1253-1258.

3. Robertson CS, Zager EL, Narayan RK, et al. Clinical evaluation of a portable near-infrared device for detection of traumatic intracranial hematomas. *J Neurotrauma*. 2010;27(9):1597-1604.

4. Strangman GE, Li Z, Zhang Q. Depth sensitivity and source-detector separations for near infrared spectroscopy based on the Colin27 brain template. *PLoS One*. 2013;8(8):e66319.

5. Strangman GE, Zhang Q, Li Z. Scalp and skull influence on near infrared photon propagation in the Colin27 brain template. *Neuroimage*. 2014;85 Pt 1:136-149.

6. Moreira-Gonzalez A, Papay FE, Zins JE. Calvarial thickness and its relation to cranial bone harvest. *Plast Reconstr Surg*. 2006;117(6):1964-1971.

7. Robertson CS, Gopinath SP, Chance B. USE OF NEAR INFRARED SPECTROSCOPY TO IDENTIFY TRAUMATIC INTRACRANIAL HEMATOMAS. https://www.spiedigitallibrary.org/journals/Journal-of-Biomedical-Optics

8. Brogan RJ, Kontojannis V, Garara B, Marcus HJ, Wilson MH. Near-infrared spectroscopy (NIRS) to detect traumatic intracranial haematoma: A systematic review and meta-analysis. *Brain Inj*. 2017;31(5):581-588.

9. Sen AN, Gopinath SP, Robertson CS. Clinical application of near-infrared spectroscopy in patients with traumatic brain injury: a review of the progress of the field. *Neurophotonics*. 2016;3(3):031409.

10. Seidel G, Cangür H, Albers T, Meyer-Wiethe K. Transcranial sonographic monitoring of hemorrhagic transformation in patients with acute middle cerebral artery infarction. *J Neuroimaging*. 2005;15(4):326-330.

11. Hakim SM, Abdellatif AA, Ali MI, Ammar MA. Reliability of transcranial sonography for assessment of brain midline shift in adult neurocritical patients: a systematic review and meta-analysis. *Minerva Anestesiol*. 2021;87(4):467-475.

12. Seidel G, Gerriets T, Kaps M, Missler U. Dislocation of the third ventricle due to space-occupying stroke evaluated by transcranial duplex sonography. *J Neuroimaging*. 1996;6(4):227-230.

13. Becker G, Krone A, Koulis D, et al. Reliability of transcranial colour-coded real-time sonography in assessment of brain tumours: correlation of ultrasound, computed tomography and biopsy findings. *Neuroradiology*. 1994;36(8):585-590.

14. Becker G, Winkler J, Bogdahn U. [Transcranial color-coded real time sonography in adults. Part 2: Cerebral hemorrhage and tumors]. *Ultraschall Med*. 1991;12(5):211-217.

15. van Leyen K, Klötzsch C, Harrer JU. Brain tumor imaging with transcranial sonography: state of the art and review of the literature. *Ultraschall Med*. 2011;32(6):572-581.
